# Genetic and Modifiable Risk Factors for Postoperative Complications of Total Joint Arthroplasty: A Genome-Wide Association and Mendelian Randomization Study

**DOI:** 10.3390/bioengineering11080797

**Published:** 2024-08-07

**Authors:** Sijia Guo, Jiping Zhang, Huiwu Li, Cheng-Kung Cheng, Jingwei Zhang

**Affiliations:** 1 School of Biomedical Engineering, Shanghai Jiao Tong University, Shanghai 200030, China; guosijia@sjtu.edu.cn (S.G.); zjp_gomm@sjtu.edu.cn (J.Z.); 2 Engineering Research Center of Digital Medicine of the Ministry of Education, Shanghai Jiao Tong University, Shanghai 200030, China; 3 Department of Orthopaedics, Ninth People’s Hospital, Shanghai Jiao Tong University School of Medicine, 639 Zhizaoju Road, Shanghai 200011, China; huiwu1223@163.com

**Keywords:** genome-wide association study, mechanical complications, Mendelian randomization, periprosthetic joint infection, total joint arthroplasty

## Abstract

**Background**: Total joint arthroplasty (TJA) is an orthopedic procedure commonly used to treat damaged joints. Despite the efficacy of TJA, postoperative complications, including aseptic prosthesis loosening and infections, are common. Moreover, the effects of individual genetic susceptibility and modifiable risk factors on these complications are unclear. This study analyzed these effects to enhance patient prognosis and postoperative management. **Methods**: We conducted an extensive genome-wide association study (GWAS) and Mendelian randomization (MR) study using UK Biobank data. The cohort included 2964 patients with mechanical complications post-TJA, 957 with periprosthetic joint infection (PJI), and a control group of 398,708 individuals. Genetic loci associated with postoperative complications were identified by a GWAS analysis, and the causal relationships of 11 modifiable risk factors with complications were assessed using MR. **Results**: The GWAS analysis identified nine loci associated with post-TJA complications. Two loci near the *PPP1R3B* and *RBM26* genes were significantly linked to mechanical complications and PJI, respectively. The MR analysis demonstrated that body mass index was positively associated with the risk of mechanical complications (odds ratio [OR]: 1.42; *p* < 0.001). Higher educational attainment was associated with a decreased risk of mechanical complications (OR: 0.55; *p* < 0.001) and PJI (OR: 0.43; *p* = 0.001). Type 2 diabetes was suggestively associated with mechanical complications (OR, 1.18, *p* = 0.02), and hypertension was suggestively associated with PJI (OR, 1.41, *p* = 0.008). Other lifestyle factors, including smoking and alcohol consumption, were not causally related to postoperative complications. **Conclusions**: The genetic loci near *PPP1R3B* and *RBM26* influenced the risk of post-TJA mechanical complications and infections, respectively. The effects of genetic and modifiable risk factors, including body mass index and educational attainment, underscore the need to perform personalized preoperative assessments and the postoperative management of surgical patients. These results indicate that integrating genetic screening and lifestyle interventions into patient care can improve the outcomes of TJA and patient quality of life.

## 1. Introduction

Total joint arthroplasty (TJA) is the standard treatment for end-stage joint disorders and can greatly improve the quality of life of and reduce pain in affected individuals [1,2]. The prevalence of end-stage joint diseases, such as osteoarthritis, is increasing globally due to an aging population and rising obesity rates. In the United States alone, the prevalence of knee osteoarthritis is estimated to be 37.4% in adults aged 60 years and older [3]. Consequently, the demand for TJA is growing, with projections indicating that by 2030, the number of total hip and knee arthroplasties will reach 572,000 and 3.48 million, respectively [4].

Despite its widespread use, TJA is associated with complications, including aseptic prosthesis loosening (APL), osteolysis, periprosthetic fractures, and periprosthetic joint infection (PJI). The prevalence of these complications varies, with studies reporting rates of 1.9% for PJI [5], 4.4% for periprosthetic fractures [6], and 7.5% for APL [7] following total hip arthroplasty. These complications can severely impact patient recovery and may require complex revision surgeries. Therefore, the early identification of individuals at a high risk of complications is crucial for implementing pre-emptive measures to reduce medical costs and improve outcomes.

The risk of developing complications after TJA is influenced by surgical techniques, implant characteristics, and a range of individual factors [8,9]. Among these, genetic factors are increasingly recognized as crucial determinants. For instance, familial aggregation analyses have shown that the risk of PJI is higher among the close relatives of affected individuals [10]. Moreover, genome-wide association studies (GWASs) identified several genetic risk loci associated with postoperative complications such as osteolysis [11]. However, achieving a genome-wide significance level is challenging because of small sample sizes and variations in hereditary factors across populations.

Lifestyle and genetic factors can affect the outcomes of TJA. Although observational studies have suggested associations between lifestyle factors and TJA outcomes [12,13,14,15,16], these associations are affected by confounding and reverse causation. Mendelian randomization (MR) uses genetic variants as instrumental variables to explain causal relationships between exposure and outcome. These variants are randomly distributed at conception and unaffected by environmental and other confounding factors [17,18], making MR analysis useful for evaluating causal associations between lifestyle factors and postoperative complications in orthopedics.

This study performed a GWAS of UK Biobank data to identify genetic markers causally associated with postoperative complications of TJA and conducted a two-sample MR analysis to investigate the causal relationships of modifiable risk factors with postoperative complications. These analyses can help guide personalized treatments and increase the efficacy of surgical interventions to improve patient care and surgical outcomes.

## 2. Materials and Methods

### 2.1. Study Design

**Figure 1:**
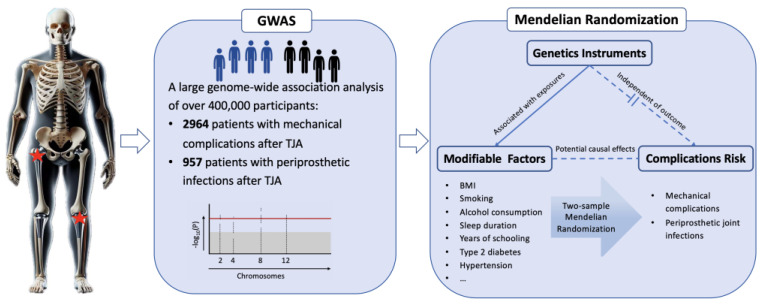
Overview of this study’s design.

This study employed a two-stage analysis consisting of a GWAS and MR (Figure 1). In the first stage, a GWAS was performed to identify genetic variants associated with postoperative complications following TJA. The second stage involved an MR analysis to investigate the causal relationship between modifiable risk factors and post-TJA complications. By combining these two powerful analytical approaches, we aimed to provide a comprehensive understanding of the genetic and modifiable risk factors contributing to complications after TJA.

### 2.2. Cohort and Phenotypes

The UK Biobank is a large-scale biomedical database of over 500,000 participants aged 40–69 years who enrolled between 2006 and 2010. In-depth genetic and health information from these participants is available to investigators for research purposes. DNA extraction and quality control were performed as described on the UK Biobank website (https://www.ukbiobank.ac.uk/) (accessed on 1 May 2024). This study was approved by the National Health Service Research Ethics Committee (Approval No. 11/NW/0382). For this research, we were granted access to genetic information from the UK Biobank (Grant No. 93966).

The inclusion criterion was patients who underwent primary TJA surgery (mainly on the hip or knee) and developed postoperative complications in internal joint prostheses. Complications were categorized into two groups: (i) mechanical complications in the internal joint prosthesis and (ii) PJI. Mechanical complications were identified using the International Classification of Diseases 10th Revision (ICD-10) code T84.0. PJI was identified using the ICD-10 code T84.5, indicating infections and inflammatory responses in the internal joint prosthesis. The control group included participants who had not undergone primary TJA, individuals who did not require revision surgery after TJA, and subjects with no postsurgical complications.

### 2.3. Statistical Analysis

#### 2.3.1. Baseline Statistics

Continuous variables were analyzed using Student’s *t*-test and were presented as medians and interquartile ranges. Categorical variables were analyzed using the chi-square test and were presented as ratios or percentages.

#### 2.3.2. GWAS Analysis

The GWAS was performed using REGENIE version 2.2 [19], and the analysis was conducted in two steps. The first step entailed fitting a genome-wide regression model using a selective subset of genetic markers to determine most of the phenotypic variations attributable to genetic factors. Quality control criteria for the genotypic variations involved single nucleotide polymorphisms (SNPs) with INFO scores < 0.1, minor allele frequencies < 0.01, genotype missing rates < 0.1, individual missing rates < 0.1, and SNPs that passed the Hardy–Weinberg equilibrium test (*p* > 1 × 10^−15^) [20]. Additionally, individuals of non-White British ancestry, individuals related to at least one cohort participant (with genetic relationship values exceeding the threshold of 0.025 in the genetic relationship matrix), and individuals who did not meet the above quality control criteria were excluded using the methods described previously [20]. A total of 509,485 variants were included in the analysis. The second step involved conducting association studies with the inferred genetic markers (7,644,147 variants). The regression models were adjusted for age, sex, body mass index (BMI), and the ten most significant population principal components.

#### 2.3.3. Functional Mapping and Annotation

The GWAS results were annotated and analyzed using the FUMA platform (http://fuma.ctglab.nl/) (accessed on 1 May 2024) [21]. The genome-wide significance for SNPs was set at *p* < 5 × 10^−8^ [22]. To balance true associations with false positives, SNPs with *p* > 1 × 10^−6^ were considered suggestively significant [11,23]. SNPs with a linkage disequilibrium (LD) r^2^ value of less than 0.6 were deemed independent. A genomic locus was defined as a region containing one or more independent significant SNPs, where the maximum distance between adjacent LD blocks of independent significant SNPs was set to 250 kb. This distance threshold was chosen to merge closely located signals into a single genomic locus, accounting for the typical extent of LD in the human genome.

An integrated methodology involving positional mapping and MAGMA gene-based mapping was used to determine the distance between genes and SNPs and the cumulative effect of multiple variants within each gene and to perform a thorough and accurate assessment of genotype–phenotype associations. In the positional mapping approach, a gene was considered to be linked to a SNP if the physical distance between them was less than 10 kb, allowing for the identification of genetic variants located close to genes and the determination of the potential functions of these variants. In the gene-based approach, we combined genome-wide SNPs within each of the 19,002 protein-coding genes, allowing the development of a gene-level test to assess the collective association between the genotypes of all the SNPs mapped to that gene and the phenotype of interest. The genome-wide significance was set at *p* < 2.63 × 10^−6^ (0.05/19,002) to account for the large number of genes tested and reduce the false-positive rate.

#### 2.3.4. MR

The causal relationships between modifiable risk factors and postoperative complications were assessed using two-sample MR and inverse variance weighting [24]. The following modifiable risk factors were analyzed: BMI, waist-to-hip ratio, smoking, alcohol consumption, sleep duration, sleep disorders, years of education, type 2 diabetes, hypertension, and major depression. The GWAS summary data for each factor were sourced from large studies on populations of European ancestry [25,26,27,28,29,30,31,32], avoiding overlap with UK Biobank data. Detailed information on the GWAS summary used for each lifestyle factor is shown in Table A1. Instrumental variables (IVs) were screened to select those strongly correlated with exposure and those not affected by confounders and to ensure that there was no genetic pleiotropy, i.e., the effects of IVs on the outcome were mediated solely by the exposure [33]. The strength of SNPs was evaluated using F statistics, and IVs with F < 10 and *p* > 5 × 10^−8^ were excluded to avoid weak instrument bias. IVs associated with potential confounders were excluded using PhenoScanner version 2 [34] (http://www.phenoscanner.medschl.cam.ac.uk/) (accessed on 1 May 2024). SNPs were clustered using a stringent criterion, and those with a high LD were excluded to ensure SNP independence.

A sensitivity analysis of the MR results was performed using MR-Egger regression, a weighted median estimator [35], and the MR pleiotropy residual sum and outlier (MR-PRESSO) method [36]. Significance thresholds were determined using Bonferroni correction. *p*-values of less than 0.004 (0.05/11) were considered statistically significant, and *p*-values between 0.004 and 0.05 were considered suggestively significant. Analyses were performed using the “TwoSampleMR” and “MR-PRESSO” packages in R software version 4.2.2.

## 3. Results

### 3.1. Baseline Characteristics

The baseline characteristics of the patients and controls are shown in Table 1 and Table A2. The average age and BMI in the control cohort were 58 years and 26.7 kg/m^2^, and 54% were men. The average age and BMI in the group with mechanical complications were 63 years and 28.9 kg/m^2^ (*p* < 0.001 vs. control), and the percentage of men was 44% (*p* = 0.011 vs. control). The average age and BMI in the group with PJI were 63 years and 29.8 kg/m^2^, and the percentage of men was 46% (all at *p* < 0.001 vs. control).

### 3.2. GWAS Analysis of Mechanical Complications after TJA

**Figure 2:**
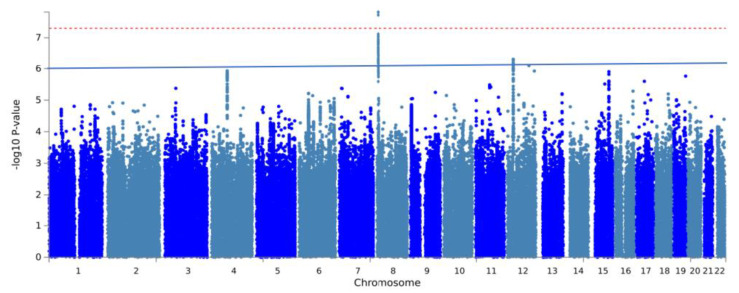
Manhattan plot of GWAS on mechanical complications after total joint arthroplasty. The red line and blue line represent genome-wide significance (*p* < 5 × 10^−8^) and suggestive significance (*p* < 1 × 10^−6^), respectively.

**Figure A1:**
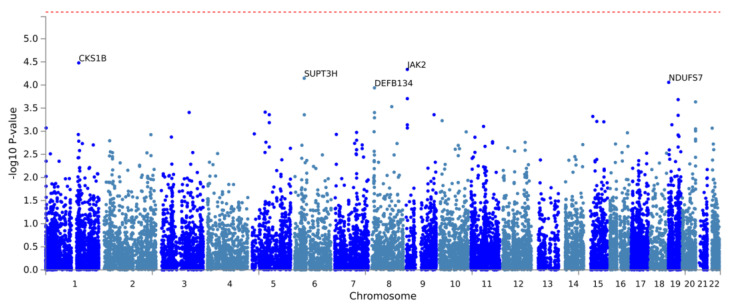
Manhattan plot of the gene-based analysis of mechanical complications. The red line indicates genome-wide significance (*p* < 2.63 × 10^−6^).

The GWAS analysis of mechanical complications identified seven independent, suggestively significant SNPs across three risk loci (*p* < 1 × 10^−6^, Figure 2, Table 2). Two of these SNPs reached genome-wide significance (*p* < 5 × 10^−8^). The most significant locus was located near the *PPP1R3B* gene at 8p23.1, containing four independent SNPs with suggestive significance: rs2929469 (G allele; odds ratio [OR], 0.86; *p* = 1.56 × 10^−8^), 8:9078016_TC_T (T allele; OR, 1.19; *p* = 1.53 × 10^−8^), rs2929305 (A allele; OR, 1.15, *p* = 1.25 × 10^−8^), and rs534523 (G allele; OR, 1.14; *p* = 3.80 × 10^−7^). Another risk locus was located at 12p11.22, with two independent SNPs: rs61916472 (A allele; OR, 0.84; *p* = 4.81 × 10^−7^) and rs2929305 (A allele; OR, 0.85; *p* = 5.12 × 10^−7^). Additionally, a significant independent SNP was identified at the 12q23.1 region near the ANO4 gene: rs772483280 (C allele; OR, 0.74; *p* = 8.05 × 10^−8^). The gene-level association analysis using MAGMA indicated that none of the genes reached genome-wide significance, and the five most significant genes were *IGFBP7*, *CTSC*, *RBM26*, *ITPA*, and *TMEM198* (Figure A1).

### 3.3. GWAS Analysis of PJI after TJA

**Figure 3:**
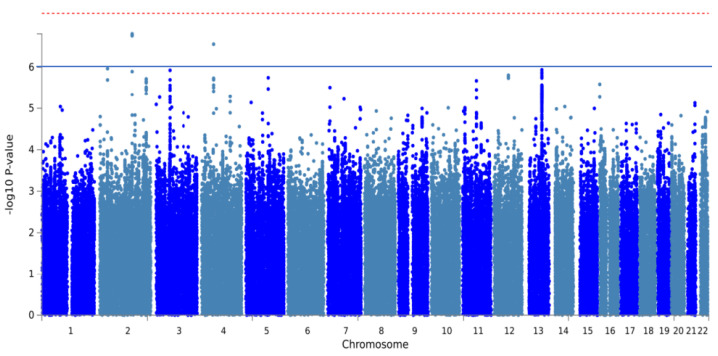
Manhattan plot of GWAS analysis of periprosthetic joint infections after total joint arthroplasty. The red line and blue line represent genome-wide significance (*p* < 5 × 10^−8^) and suggestive significance (*p* < 1 × 10^−6^), respectively.

**Figure A2:**
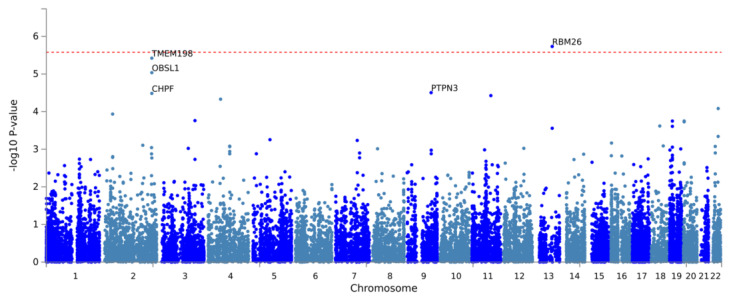
Manhattan plot of the gene-based analysis of periprosthetic joint infections. The red line represents genome-wide significance (*p* < 2.63 × 10^−6^).

The GWAS analysis of 957 individuals with PJI identified two independent SNPs across two risk loci. Both SNPs achieved suggestive genome-wide significance (*p* < 1 × 10^−6^, Figure 3, Table 3). The most significant locus was located near the *PRPF40A* gene at 2q23.2, featuring the independent, suggestively significant SNP rs187929349 (T allele; OR, 1.19; *p* = 1.30 × 10^−7^). Another significant locus was located at 4q12, with the independent, suggestively significant SNP rs761716701 (T allele; OR, 1.19; *p* = 7.70 × 10^−8^). The MAGMA analysis showed that *RBM26* reached genome-wide significance (*p* < 2.63 × 10^−8^, Figure A2).

### 3.4. Causal Relationships of Modifiable Risk Factors with Mechanical Complications after TJA

**Figure 4:**
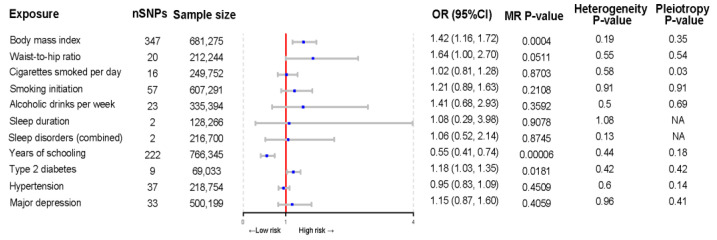
Forest plot of Mendelian randomization estimates of the association of modifiable lifestyle factors with mechanical complications after total joint arthroplasty. MR, Mendelian randomization; OR, odds ratio; CI, confidence interval.

The MR analysis of the causal relationship of 11 modifiable risk factors with mechanical complications after TJA is shown in Figure 4. BMI and years of education were strongly associated with the risk of mechanical complications (OR per standard deviation decrease in BMI = 1.42; 95% confidence interval (CI), 1.16–1.72; *p* = 0.0004; OR per year increase in education = 0.55; 95% CI, 0.41–0.74; *p* = 6.85 × 10^−5^). Moreover, type 2 diabetes showed a suggestive significant association with the risk of mechanical complications before correction for multiple comparisons (OR, 1.18; 95% CI, 1.03–1.35, *p* = 0.02). Sensitivity analyses demonstrated the robustness of these findings, with no evidence of heterogeneity or pleiotropy. Waist-to-hip ratio, smoking, alcohol intake, sleep duration, sleep disorders, hypertension, and depression were not causally related to the risk of mechanical complications.

### 3.5. Causal Associations of Modifiable Risk Factors with PJI after TJA

**Figure 5:**
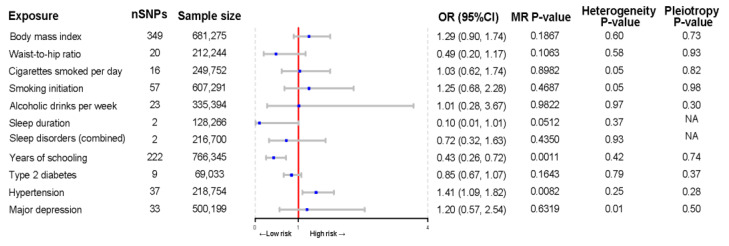
Forest plot of Mendelian randomization estimates of the association of modifiable lifestyle factors with periprosthetic joint infections after total joint arthroplasty. MR, Mendelian randomization; OR, odds ratio; CI, confidence interval.

The MR analysis of the causal relationship of modifiable risk factors with PJI after TJA is summarized in Figure 5. Genetically predicted years of schooling were associated with a decreased risk of PJI (OR per year increase in education = 0.43; 95% CI, 0.26–0.72; *p* = 0.001). In contrast, hypertension was suggestively associated with the risk of PJI (OR, 1.41; 95% CI, 1.09–1.82; *p* = 0.008). The results of sensitivity analyses showed no horizontal pleiotropy or heterogeneity. In addition, BMI, waist-to-hip ratio, smoking, alcohol consumption, sleep duration, sleep disorders, type 2 diabetes mellitus, and depression were not causally related to the risk of PJI.

## 4. Discussion

Arthroplasty is becoming more common with the increasing prevalence of end-stage joint diseases in aging populations [37]. Despite advancements in surgical techniques, implant materials and design, and the use of teleoperated surgical robots, postoperative complications pose significant clinical challenges [38,39], and several patients have postsurgical complications because of individual factors [40], highlighting the importance of identifying the genetic and modifiable risk factors to improve surgical outcomes and prognosis.

Our analysis of genetic data from over 400,000 UK Biobank participants identified nine genetic risk loci associated with mechanical complications and PJI after TJA. Of the seven loci related to mechanical complications, two loci located near the *PPP1R3B* and *RBM26* genes at 8p23.1 reached genome-wide significance. The *PPP1R3B* gene encodes the regulatory subunit of protein phosphatase 1 (PP1), which is mainly expressed in the liver, skeletal muscle, and cardiac tissues. The role of PP1 in the complications of TJA is unknown. Previous GWASs have found that *PPP1R3B* variants affect bone mineral density (BMD) [41,42,43] and genetic susceptibility to osteoarthritis [44]. Moreover, *PPP1R3B* may regulate BMD by modulating osteoclast and osteoblast activity, ultimately increasing the risk of APL, osteolysis, and periprosthetic fractures in patients with TJA. In turn, *PPP1R3B* polymorphisms are significantly associated with lipid and C-reactive protein levels [45] and inflammation [46], which can exacerbate periprosthetic osteolysis. The role of *PPP1R3B* in glycogen synthesis in the liver and skeletal muscle [47,48,49] suggests that this gene may affect patient recovery and the rate of postoperative complications However, further research is needed to determine the role of *PPP1R3B* in mechanical complications after TJA.

Our findings showed that the *RBM26* gene was significantly associated with PJI. RBM26 is an RNA-binding motif protein involved in the nuclear turnover of polyadenylated RNA in mammalian cells. *RMB26* is implicated in several biological processes and diseases, including osteopetrosis and anthracosis [50]. These associations suggest that RBM26 may play a role in inflammatory pathways, which are known risk factors for postoperative infections. Additionally, *RBM26* is also involved in RNA splicing and processing [50]. Surgical trauma induces cellular stress and physiological and immune responses, which involve gene expression and regulation and RNA splicing and processing. Therefore, *RBM26* may influence responses to surgical trauma. These responses are essential for the rapid and efficient clearance of pathogens from the surgical site to reduce the risk of infections. Moreover, *RBM4*, an *RBM26* homolog, is highly expressed in skeletal and cardiac muscle and mediates inflammation by regulating the transcription and alternative splicing of inflammatory genes in HeLa cells, suggesting that *RBM26* regulates inflammation in skeletal muscle [51]. Since the skeletal muscle is affected by TJA, the alterations in RNA processing caused by *RBM26* can affect tissue regeneration and inflammatory responses after surgery. Hypothetically, if *RBM26* regulates the expression of immune and cytokine genes, then variants in *RBM26* may affect immune responses during or after TJA. This mechanism may also explain the genetic susceptibility to PJI in some patients. Nonetheless, further studies are necessary to elucidate the pathways by which *RBM26* influences PJI after TJA.

Several risk factors associated with postoperative complications have been identified, including obesity [52,53], smoking [54,55], alcohol consumption [56,57], diabetes [15,58], and hypertension [16]. However, most of these associations were derived from observational studies, which are inherently affected by confounders and biases. This study evaluated these factors using GWAS and MR analyses to estimate causal effects. The MR results demonstrated that BMI, educational attainment, diabetes, and hypertension were causally related to postoperative complications. Nonetheless, the results of observational studies on the effect of BMI on the postoperative complications of TJA are conflicting [13,53,59,60]. BMI positively correlates with tensile stress in the joint prosthesis, increasing prosthesis wear and the incidence of APL. In this respect, a biomechanical study [61] has shown that BMI is positively linked with tensile strain in the distal femur and micromotion between the prosthesis and the femur. Moreover, BMI negatively correlates with prosthesis stability during gait. These results are consistent with our genetic analysis results, which showed that BMI was positively associated with the risk of postoperative mechanical complications. In turn, educational levels were negatively correlated with complication rates, suggesting that socioeconomic factors influence postsurgical outcomes, which is consistent with health data demonstrating that higher education is correlated with healthier lifestyles [62], potentially mitigating inherent risk factors for postoperative complications.

This study also assessed the effects of type 2 diabetes and hypertension, which are risk factors for postoperative complications [16,63,64], and showed that type 2 diabetes was a causal factor for mechanical complications, and hypertension was linked with an increased risk of PJI. These findings corroborate the evidence from several recent studies [65,66,67]. Several potential pathogenetic mechanisms may explain these associations. Diabetes can impair bone metabolism and healing, leading to decreased bone mineral density, increased fracture risk [68,69], and compromised mechanical properties of the bone–implant interface due to hyperglycemia-induced collagen fiber dysfunction [70] and the accumulation of advanced glycation end products (AGEs) [71,72]. Hypertension may cause endothelial dysfunction, impaired microvascular circulation, and reduced tissue perfusion and oxygenation [73], hindering the immune response and increasing susceptibility to infection. Elevated oxidative stress and the pro-inflammatory state associated with hypertension could facilitate bacterial colonization and infection [74,75], while metabolic dysregulation may impede wound healing and immune function [76]. However, these proposed mechanisms are based on existing literature and require further validation through targeted research to elucidate the precise biological pathways linking these conditions to postoperative complications.

This study has several strengths. First, the study performed one of the most thorough GWAS analyses on post-TJA complications to date. Compared with previous GWASs [11,23], our analyses identified more risk loci and improved the understanding of genetic risk factors for these complications. In addition, using a control group and a large-scale GWAS dataset provided substantial statistical power to estimate the true causal relationships of modifiable risk factors with postoperative complications.

This study has several limitations. First, the observational nature of the UK Biobank data prevented the detailed analysis of surgical variables and their association with the time of postoperative complications; thus, we could not eliminate bias from surgical factors. The absence of standardized care across the patients included in the study may have introduced confounding factors that influenced the identified genetic associations. Although the large sample size of our study may help to attenuate the impact of these confounding variables, it is essential to recognize that the association between complications after TJA and the isolated genes we discovered requires further validation. Third, UK Biobank databases have a smaller number of TJA patients than GWAS databases. This limitation was addressed by increasing the detection rate using a less stringent threshold (*p* < 1 × 10^−6^) for suggestive significance and increasing the control group by including patients who did not undergo TJA surgery. However, this approach can potentially increase the false-positive rate. Fourth, our findings are preliminary because we did not include a replication cohort. Fifth, the MR analyses included only common modifiable risk factors because of the limited availability of genomic datasets. Sixth, the MR did not estimate nonlinear causal relationships [77], such as those between BMI and PJI; thus, the absence of relationships should be interpreted with caution. Seventh, the results cannot be generalized to other populations because the GWAS findings and MR analyses were based on European populations.

Future studies can address these limitations and gaps by (1) including larger cohorts, particularly in the case group, to improve the statistical power, discovery rate, and reliability of GWAS findings, (2) incorporating more surgical variables to understand their impact on genetic susceptibility to postoperative complications, (3) evaluating other modifiable risk factors, and (4) validating these findings across populations. Furthermore, to strengthen the robustness and generalizability of the findings, future research should prioritize the inclusion of patient cohorts who have received standardized care. This approach would help to account for variations in care quality and other procedural discrepancies that may confound the identified genetic associations. Additionally, conducting well-designed, multicenter clinical trials is strongly recommended to ensure that the findings can be replicated across diverse clinical settings and patient populations.

Our findings underscore the potential clinical benefits of incorporating genetic and modifiable risk factor assessments into preoperative planning and postoperative management. For instance, preoperative genetic screening can inform the design of personalized prostheses to mitigate the risk of infections, osteolysis, and APL. Similarly, genetic and lifestyle assessments can guide postoperative care and lifestyle modifications to prevent complications. Identifying the significant causal relationships of modifiable risk factors, including BMI, education level, diabetes mellitus, and hypertension, with postoperative complications enriches our understanding of these risk factors and improves patient management and the safety and success of TJA procedures.

## 5. Conclusions

This study identified genetic factors associated with postoperative complications of TJA. GWAS and MR analyses identified crucial genetic markers and a significant causal relationship between BMI, educational levels, and the risk of mechanical complications. Two loci located near the *PPP1R3B* and *RBM26* genes were significant risk factors for mechanical complications and infections, respectively. However, we found no causal links between other modifiable risk factors and post-TJA complications.

These findings provide valuable insights for clinicians, enabling them to incorporate genetic risk assessment into preoperative planning and postoperative management. By identifying patients with high-risk genetic variants, such as those near the *PPP1R3B* and *RBM26* genes, healthcare providers can develop personalized prevention and treatment strategies to mitigate the risk of complications and improve patient outcomes. Furthermore, our results underscore the importance of addressing modifiable risk factors, such as obesity and diabetes, through early screening and targeted interventions to optimize the results of total joint arthroplasty.

## Figures and Tables

**Table 1:** Baseline characteristics.

Characteristic	Control (N = 398,708)	MC-PJ ^1^ (N = 2964)	*p*-Value ^2^	PJI ^1^ (N = 957)	*p*-Value ^2^
Sex (Male: female)	183,333:215,375	1294:1670	0.011	444:513	<0.001
Age (y)	58 (51, 63)	63 (59, 66)	<0.001	63 (58, 66)	<0.001
BMI (kg/m^2^)	26.7 (24.1, 29.8)	28.9 (26.0, 32.4)	<0.001	29.8 (26.8, 33.9)	<0.001

^1^ N (%); median (IQR). ^2^ Pearson’s Chi-squared test; Wilcoxon rank sum test. MC-PJ: Mechanical complications after primary TJA; PJI: periprosthetic joint infection.

**Table 2:** Independent, suggestively significant SNPs associated with mechanical complications after total joint arthroplasty.

Chromosome	Position	Nearest Gene	SNP	Major/Minor Allele	*p* Value	OR
**8**	9062656	*PPP1R3B*	rs2929469	T/G	1.56 × 10^−8^	0.86
**8**	9078016	*PPP1R3B*	8:9078016_TC_T	TC/T	1.53 × 10^−8^	1.19
**8**	9085217	*PPP1R3B*	rs2929305	G/A	1.25 × 10^−7^	1.15
**8**	9884999	*MSRA*	rs534523	C/G	3.80 × 10^−7^	1.14
**12**	27976657	*MANSC4*	rs61916472	G/A	4.81 × 10^−7^	0.84
**12**	27986940	*MANSC4*	rs10843003	T/G	5.12 × 10^−7^	0.85
**12**	101231042	*ANO4*	rs772483280	CAACAACCATA/C	8.05 × 10^−7^	0.74

**Table 3:** Independent, suggestively significant SNPs associated with periprosthetic joint infections after total joint arthroplasty.

Chromosome	Position	Nearest Gene	SNP	Major/Minor Allele	*p* Value	OR
2	153869991	*PRPF40A*	rs187929349	C/T	1.57 × 10^−7^	1.30
4	57912498	*IGFBP7*	rs761716701	TTC/T	2.78 × 10^−7^	0.77

## Data Availability

The GWAS summary dataset involved in the study will be made publicly available after the publication of the article, and access can currently be obtained by contacting the corresponding author.

## Appendix A

**Table A1:** GWAS summary data on lifestyle factors included in Mendelian randomization.

Lifestyle Factor	Participants	IVs	Consortium	PMID
Body mass index	681,275 European	347	GIANT	30124842
Waist-to-hip ratio	212,244 European	20	GIANT	25673412
Cigarettes smoked per day	249,752 European	16	GSCAN	30643251
Smoking initiation	60,7291 European	57	GSCAN	30643251
Alcoholic drinks per week	335,394 European	23	GSCAN	30643251
Sleep duration	127,573 European	2	NA	27494321
Sleep disorders	216,700 European	2	FinnGen	NA
Years of schooling	766,345 European	222	SSGAC	30038396
Type 2 diabetes	69,033 European	9	DIAGRAM	22885922
Hypertension	218,754 European	37	FinnGen	NA
Major depression	500,199 European	33	PGC	30718901

IVs: instrumental variables.

**Table A2:** Detailed baseline characteristics.

Characteristic	Control (N = 398,708)	MC-PJ ^1^ (N = 2964)	*p*-Value ^2^	PJI ^1^ (N = 957)	*p*-Value ^2^
**Ever smoked**	239,143 (60%)	1811 (61%)	0.2	597 (63%)	0.085
**Smoking status**			<0.001		<0.001
Never	217,386 (55%)	1467 (49%)		465 (49%)	
Previous	139,814 (35%)	1236 (42%)		395 (41%)	
Current	40,133 (10%)	248 (8.4%)		89 (9.3%)	
**Pack Years of smoking**	19 (10, 32)	23 (13, 37)	<0.001	23 (13, 37)	<0.001
**Alcohol drinker status**			<0.001		<0.001
Never	12,381 (3.1%)	135 (4.6%)		41 (4.3%)	
Previous	13,468 (3.4%)	146 (4.9%)		57 (6.0%)	
Current	372,525 (93%)	2678 (90%)		858 (90%)	
**Systolic BP**	139 (127, 153)	143 (132, 158)	0.003	139 (130, 158)	0.7
**Diastolic BP**	83 (75, 90)	84 (76, 90)	0.5	84 (76, 90)	0.9
**Diabetes diagnosed by doctor**	19,001 (4.8%)	191 (6.4%)	<0.001	77 (8.0%)	<0.001
**FN BMD**	0.93 (0.84, 1.03)	0.96 (0.86, 1.02)	0.4	0.97 (0.89, 1.04)	0.3
**Heel BMD**	−0.47 (−1.15, 0.30)	−0.55 (−1.26, 0.31)	0.004	−0.40 (−1.14, 0.39)	0.5
**HOA**	18,568 (4.7%)	1223 (41%)	<0.001	404 (42%)	<0.001
**KOA**	29,568 (7.4%)	1298 (44%)	<0.001	508 (53%)	<0.001
**Osteonecrosis**	678 (0.2%)	75 (2.5%)	<0.001	20 (2.1%)	<0.001
**Infectious arthropathies**	682 (0.2%)	47 (1.6%)	<0.001	76 (7.9%)	<0.001
**Inflammatory polyarthropathies**	36,416 (9.1%)	1039 (35%)	<0.001	352 (37%)	<0.001
**Arthrosis**	71,432 (18%)	2520 (85%)	<0.001	844 (88%)	<0.001
**Systemic connective tissue disorders**	6244 (1.6%)	131 (4.4%)	<0.001	53 (5.5%)	<0.001
**Disorders of bone density and structure**	19,188 (4.8%)	511 (17%)	<0.001	166 (17%)	<0.001

^1^ N (%); median (IQR). ^2^ Pearson’s Chi-squared test; Wilcoxon rank sum test. MC-PJ: Mechanical complications after TJA; PJI: periprosthetic joint infection; BMD: bone mineral density; KOA: knee osteoarthritis; HOA: hip osteoarthritis.

## References

[1] Hawker G.A., Badley E.M., Borkhoff C.M., Croxford R., Davis A.M., Dunn S., Gignac M.A., Jaglal S.B., Kreder H.J., Sale J.E.M. (2013). Which Patients Are Most Likely to Benefit from Total Joint Arthroplasty?. Arthritis Rheum..

[2] Ferguson R.J., Palmer A.J., Taylor A., Porter M.L., Malchau H., Glyn-Jones S. (2018). Hip Replacement. Lancet.

[3] Zhang Y., Jordan J.M. (2010). Epidemiology of Osteoarthritis. Clin. Geriatr. Med..

[4] Sloan M., Premkumar A., Sheth N.P. (2018). Projected Volume of Primary Total Joint Arthroplasty in the U.S., 2014 to 2030. J. Bone Jt. Surg. Am..

[5] Izakovicova P., Borens O., Trampuz A. (2019). Periprosthetic Joint Infection: Current Concepts and Outlook. EFORT Open Rev..

[6] Pivec R., Issa K., Kapadia B.V., Cherian J.J., Maheshwari A.V., Bonutti P.M., Mont M.A. (2015). Incidence and Future Projections of Periprosthetic Femoral Fracture Following Primary Total Hip Arthroplasty: An Analysis of International Registry Data. J. Long Term Eff. Med. Implant..

[7] Ulrich S.D., Seyler T.M., Bennett D., Delanois R.E., Saleh K.J., Thongtrangan I., Kuskowski M., Cheng E.Y., Sharkey P.F., Parvizi J. (2008). Total Hip Arthroplasties: What Are the Reasons for Revision?. Int. Orthop..

[8] Cherian J.J., Jauregui J.J., Banerjee S., Pierce T., Mont M.A. (2015). What Host Factors Affect Aseptic Loosening after THA and TKA?. Clin. Orthop. Relat. Res..

[9] Edwards P.K., Mears S.C., Stambough J.B., Foster S.E., Barnes C.L. (2018). Choices, Compromises, and Controversies in Total Knee and Total Hip Arthroplasty Modifiable Risk Factors: What You Need to Know. J. Arthroplast..

[10] Anderson M.B., Curtin K., Wong J., Pelt C.E., Peters C.L., Gililland J.M. (2017). Familial Clustering Identified in Periprosthetic Joint Infection Following Primary Total Joint Arthroplasty: A Population-Based Cohort Study. J. Bone Jt. Surg. Am..

[11] MacInnes S.J., Hatzikotoulas K., Fenstad A.M., Shah K., Southam L., Tachmazidou I., Hallan G., Dale H., Panoutsopoulou K., Furnes O. (2019). The 2018 Otto Aufranc Award: How Does Genome-Wide Variation Affect Osteolysis Risk after THA?. Clin. Orthop. Relat. Res..

[12] Bourne R., Mukhi S., Zhu N., Keresteci M., Marin M. (2007). Role of Obesity on the Risk for Total Hip or Knee Arthroplasty. Clin. Orthop. Relat. Res..

[13] Zusmanovich M., Kester B.S., Schwarzkopf R. (2018). Postoperative Complications of Total Joint Arthroplasty in Obese Patients Stratified by BMI. J. Arthroplast..

[14] Teng S., Yi C., Krettek C., Jagodzinski M. (2015). Smoking and Risk of Prosthesis-Related Complications after Total Hip Arthroplasty: A Meta-Analysis of Cohort Studies. PLoS ONE.

[15] Martínez-Huedo M.A., Jiménez-García R., Jiménez-Trujillo I., Hernández-Barrera V., Del Rio Lopez B., López-de-Andrés A. (2017). Effect of Type 2 Diabetes on In-Hospital Postoperative Complications and Mortality after Primary Total Hip and Knee Arthroplasty. J. Arthroplast..

[16] Li X., Sun H., Li H., Huang Z., Chen M., Li D., Cai Z., Xu J., Ma R. (2023). Post-Operative Complications of Total Knee Arthroplasty in Patients with Hypertension. Int. Orthop..

[17] Lawlor D.A., Harbord R.M., Sterne J.A.C., Timpson N., Davey Smith G. (2008). Mendelian Randomization: Using Genes as Instruments for Making Causal Inferences in Epidemiology. Stat. Med..

[18] Davey Smith G., Hemani G. (2014). Mendelian Randomization: Genetic Anchors for Causal Inference in Epidemiological Studies. Hum. Mol. Genet..

[19] Mbatchou J., Barnard L., Backman J., Marcketta A., Kosmicki J.A., Ziyatdinov A., Benner C., O’Dushlaine C., Barber M., Boutkov B. (2021). Computationally Efficient Whole-Genome Regression for Quantitative and Binary Traits. Nat. Genet..

[20] Kulm S., Kolin D.A., Langhans M.T., Kaidi A.C., Elemento O., Bostrom M.P., Shen T.S. (2022). Characterization of Genetic Risk of End-Stage Knee Osteoarthritis Treated with Total Knee Arthroplasty: A Genome-Wide Association Study. J. Bone Jt. Surg. Am..

[21] Watanabe K., Taskesen E., Van Bochoven A., Posthuma D. (2017). Functional Mapping and Annotation of Genetic Associations with FUMA. Nat. Commun..

[22] Barsh G.S., Copenhaver G.P., Gibson G., Williams S.M. (2012). Guidelines for Genome-Wide Association Studies. PLoS Genet..

[23] Koks S., Wood D.J., Reimann E., Awiszus F., Lohmann C.H., Bertrand J., Prans E., Maasalu K., Märtson A. (2020). The Genetic Variations Associated with Time to Aseptic Loosening after Total Joint Arthroplasty. J. Arthroplast..

[24] Burgess S., Foley C.N., Zuber V. (2018). Inferring Causal Relationships between Risk Factors and Outcomes from Genome-Wide Association Study Data. Annu. Rev. Genom. Hum. Genet..

[25] Yengo L., Sidorenko J., Kemper K.E., Zheng Z., Wood A.R., Weedon M.N., Frayling T.M., Hirschhorn J., Yang J., Visscher P.M. (2018). Meta-Analysis of Genome-Wide Association Studies for Height and Body Mass Index in ~700000 Individuals of European Ancestry. Hum. Mol. Genet..

[26] Liu M., Jiang Y., Wedow R., Li Y., Brazel D.M., Chen F., Datta G., Davila-Velderrain J., 23andMe Research Team, HUNT All-In Psychiatry (2019). Association Studies of up to 1.2 Million Individuals Yield New Insights into the Genetic Etiology of Tobacco and Alcohol Use. Nat. Genet..

[27] Jones S.E., Tyrrell J., Wood A.R., Beaumont R.N., Ruth K.S., Tuke M.A., Yaghootkar H., Hu Y., Teder-Laving M., Hayward C. (2016). Genome-Wide Association Analyses in 128,266 Individuals Identifies New Morningness and Sleep Duration Loci. PLoS Genet..

[28] Morris A.P., Voight B.F., Teslovich T.M., Ferreira T., Segrè A.V., Steinthorsdottir V., Strawbridge R.J., Khan H., Grallert H., The DIAbetes Genetics Replication and Meta-Analysis (DIAGRAM) Consortium (2012). Large-Scale Association Analysis Provides Insights into the Genetic Architecture and Pathophysiology of Type 2 Diabetes. Nat. Genet..

[29] Lee J.J., Wedow R., Okbay A., Kong E., Maghzian O., Zacher M., Nguyen-Viet T.A., Bowers P., Sidorenko J., Karlsson Linnér R. (2018). Gene Discovery and Polygenic Prediction from a Genome-Wide Association Study of Educational Attainment in 1.1 Million Individuals. Nat. Genet..

[30] Zheng X., Zhou X., Tong L., Gu W., Wang S., Yuang W., Zhang C., Zhang C., Zhang C., Wan B. (2023). Mendelian Randomization Study of Gastroesophageal Reflux Disease and Major Depression. PLoS ONE.

[31] Shungin D., Winkler T.W., Croteau-Chonka D.C., Ferreira T., Locke A.E., Mägi R., Strawbridge R.J., Pers T.H., Fischer K., Justice A.E. (2015). New Genetic Loci Link Adipose and Insulin Biology to Body Fat Distribution. Nature.

[32] Kurki M.I., Karjalainen J., Palta P., Sipilä T.P., Kristiansson K., Donner K.M., Reeve M.P., Laivuori H., Aavikko M., Kaunisto M.A. (2023). FinnGen Provides Genetic Insights from a Well-Phenotyped Isolated Population. Nature.

[33] Deng M.-G., Liu F., Liang Y., Wang K., Nie J.-Q., Liu J. (2023). Association between Frailty and Depression: A Bidirectional Mendelian Randomization Study. Sci. Adv..

[34] Kamat M.A., Blackshaw J.A., Young R., Surendran P., Burgess S., Danesh J., Butterworth A.S., Staley J.R. (2019). PhenoScanner V2: An Expanded Tool for Searching Human Genotype–Phenotype Associations. Bioinformatics.

[35] Bowden J., Davey Smith G., Haycock P.C., Burgess S. (2016). Consistent Estimation in Mendelian Randomization with Some Invalid Instruments Using a Weighted Median Estimator. Genet. Epidemiol..

[36] Verbanck M., Chen C.-Y., Neale B., Do R. (2018). Detection of Widespread Horizontal Pleiotropy in Causal Relationships Inferred from Mendelian Randomization between Complex Traits and Diseases. Nat. Genet..

[37] Kurtz S., Ong K., Lau E., Mowat F., Halpern M. (2007). Projections of Primary and Revision Hip and Knee Arthroplasty in the United States from 2005 to 2030. J. Bone Jt. Surg. Am..

[38] Talmo C.T., Aghazadeh M., Bono J.V. (2012). Perioperative Complications Following Total Joint Replacement. Clin. Geriatr. Med..

[39] George J., Chughtai M., Khlopas A., Klika A.K., Barsoum W.K., Higuera C.A., Mont M.A. (2018). Readmission, Reoperation, and Complications: Total Hip vs Total Knee Arthroplasty. J. Arthroplast..

[40] Santaguida P.L., Hawker G.A., Hudak P.L., Glazier R., Mahomed N.N., Kreder H.J., Coyte P.C., Wright J.G. (2008). Patient Characteristics Affecting the Prognosis of Total Hip and Knee Joint Arthroplasty: A Systematic Review. Can. J. Surg..

[41] Kemp J.P., Morris J.A., Medina-Gomez C., Forgetta V., Warrington N.M., Youlten S.E., Zheng J., Gregson C.L., Grundberg E., Trajanoska K. (2017). Identification of 153 New Loci Associated with Heel Bone Mineral Density and Functional Involvement of GPC6 in Osteoporosis. Nat. Genet..

[42] Medina-Gomez C., Kemp J.P., Trajanoska K., Luan J., Chesi A., Ahluwalia T.S., Mook-Kanamori D.O., Ham A., Hartwig F.P., Evans D.S. (2018). Life-Course Genome-Wide Association Study Meta-Analysis of Total Body BMD and Assessment of Age-Specific Effects. Am. J. Hum. Genet..

[43] Morris J.A., Kemp J.P., Youlten S.E., Laurent L., Logan J.G., Chai R.C., Vulpescu N.A., Forgetta V., Kleinman A., 23andMe Research Team (2019). An Atlas of Genetic Influences on Osteoporosis in Humans and Mice. Nat. Genet..

[44] Zengini E., Hatzikotoulas K., Tachmazidou I., Steinberg J., Hartwig F.P., Southam L., Hackinger S., Boer C.G., Styrkarsdottir U., Gilly A. (2018). Genome-Wide Analyses Using UK Biobank Data Provide Insights into the Genetic Architecture of Osteoarthritis. Nat. Genet..

[45] Zhang Y., Gan W., Tian C., Li H., Lin X., Chen Y. (2013). Association of PPP1R3B polymorphisms with blood lipid and C-reactive protein levels in a Chinese population (PPP1R3B C). J. Diabetes.

[46] Dehghan A., Dupuis J., Barbalic M., Bis J.C., Eiriksdottir G., Lu C., Pellikka N., Wallaschofski H., Kettunen J., Henneman P. (2011). Meta-Analysis of Genome-Wide Association Studies in >80,000 Subjects Identifies Multiple Loci for C-Reactive Protein Levels. Circulation.

[47] Migocka-Patrzałek M., Elias M. (2021). Muscle Glycogen Phosphorylase and Its Functional Partners in Health and Disease. Cells.

[48] Stender S., Smagris E., Lauridsen B.K., Kofoed K.F., Nordestgaard B.G., Tybjærg-Hansen A., Pennacchio L.A., Dickel D.E., Cohen J.C., Hobbs H.H. (2018). Relationship between Genetic Variation at PPP1R3B and Levels of Liver Glycogen and Triglyceride. Hepatology.

[49] Creasy K.T., Mehta M.B., Park J., Schneider C.V., Shewale S.V., Millar J.S., Hand N.J., Baur J.A., Rader D.J. (2023). PPP1R3B Is a Metabolic Switch That Shifts Hepatic Energy Storage from Lipid to Glycogen. bioRxiv.

[50] Safran M., Dalah I., Alexander J., Rosen N., Iny Stein T., Shmoish M., Nativ N., Bahir I., Doniger T., Krug H. (2010). GeneCards Version 3: The Human Gene Integrator. Database.

[51] Wang W.-Y., Quan W., Yang F., Wei Y.-X., Chen J.-J., Yu H., Xie J., Zhang Y., Li Z.-F. (2020). RBM4 Modulates the Proliferation and Expression of Inflammatory Factors via the Alternative Splicing of Regulatory Factors in HeLa Cells. Mol. Genet. Genom..

[52] Haynes J., Nam D., Barrack R.L. (2017). Obesity in Total Hip Arthroplasty: Does It Make a Difference?. Bone Jt. J..

[53] Kerkhoffs G.M.M.J., Servien E., Dunn W., Dahm D., Bramer J.A.M., Haverkamp D. (2012). The Influence of Obesity on the Complication Rate and Outcome of Total Knee Arthroplasty: A Meta-Analysis and Systematic Literature Review. J. Bone Jt. Surg. Am..

[54] Singh J.A. (2011). Smoking and Outcomes after Knee and Hip Arthroplasty: A Systematic Review. J. Rheumatol..

[55] Duchman K.R., Gao Y., Pugely A.J., Martin C.T., Noiseux N.O., Callaghan J.J. (2015). The Effect of Smoking on Short-Term Complications Following Total Hip and Knee Arthroplasty. J. Bone Jt. Surg. Am..

[56] Rotevatn T.A., Bøggild H., Olesen C.R., Torp-Pedersen C., Mortensen R.N., Jensen P.F., Overgaard C. (2017). Alcohol Consumption and the Risk of Postoperative Mortality and Morbidity after Primary Hip or Knee Arthroplasty—A Register-Based Cohort Study. PLoS ONE.

[57] Horn A.R., Diamond K.B., Ng M.K., Vakharia R.M., Mont M.A., Erez O. (2021). The Association of Alcohol Use Disorder with Perioperative Complications Following Primary Total Hip Arthroplasty. Hip Pelvis..

[58] Han H.-S., Kang S.-B. (2013). Relations between Long-Term Glycemic Control and Postoperative Wound and Infectious Complications after Total Knee Arthroplasty in Type 2 Diabetics. Clin. Orthop. Surg..

[59] Baker P., Petheram T., Jameson S., Reed M., Gregg P., Deehan D. (2012). The Association Between Body Mass Index and the Outcomes of Total Knee Arthroplasty. J. Bone Jt. Surg. Am..

[60] Burn E., Edwards C.J., Murray D.W., Silman A., Cooper C., Arden N.K., Prieto-Alhambra D., Pinedo-Villanueva R. (2019). The Impact of BMI and Smoking on Risk of Revision Following Knee and Hip Replacement Surgery: Evidence from Routinely Collected Data. Osteoarthr. Cartil..

[61] Wan Q., Zhang A., Liu Y., Chen H., Zhang J., Xue H., Han Q., Wang J. (2023). The Influence of Body Weight Index on Initial Stability of Uncemented Femoral Knee Protheses: A Finite Element Study. Heliyon.

[62] Puka K., Buckley C., Mulia N., Lasserre A.M., Rehm J., Probst C. (2022). Educational Attainment and Lifestyle Risk Factors Associated With All-Cause Mortality in the US. JAMA Health Forum.

[63] Moon H.K., Han C.D., Yang I.H., Cha B.S. (2008). Factors Affecting Outcome after Total Knee Arthroplasty in Patients with Diabetes Mellitus. Yonsei Med. J..

[64] Triantafyllopoulos G.K., Soranoglou V.G., Memtsoudis S.G., Sculco T.P., Poultsides L.A. (2018). Rate and Risk Factors for Periprosthetic Joint Infection among 36,494 Primary Total Hip Arthroplasties. J. Arthroplast..

[65] Muffly B.T., Ayeni A.M., Bonsu J.M., Heo K., Premkumar A., Guild G.N. (2024). Early versus Late Periprosthetic Joint Infection after Total Knee Arthroplasty: Do Patient Differences Exist?. J. Arthroplast..

[66] Lucenti L., Testa G., Caldaci A., Sammartino F., Cicio C., Ilardo M., Sapienza M., Pavone V. (2024). Preoperative Risk Factors for Periprosthetic Joint Infection: A Narrative Review of the Literature. Healthcare.

[67] Deng Y., Smith P.N., Li R.W. (2023). Diabetes Mellitus Is a Potential Risk Factor for Aseptic Loosening around Hip and Knee Arthroplasty. BMC Musculoskelet. Disord..

[68] Napoli N., Chandran M., Pierroz D.D., Abrahamsen B., Schwartz A.V., Ferrari S.L., IOF Bone and Diabetes Working Group (2017). Mechanisms of Diabetes Mellitus-Induced Bone Fragility. Nat. Rev. Endocrinol..

[69] Vestergaard P. (2007). Discrepancies in Bone Mineral Density and Fracture Risk in Patients with Type 1 and Type 2 Diabetes—A Meta-Analysis. Osteoporos. Int..

[70] Retzepi M., Donos N. (2010). The Effect of Diabetes Mellitus on Osseous Healing. Clin. Oral. Implant. Res..

[71] Franke S., Rüster C., Pester J., Hofmann G., Oelzner P., Wolf G. (2011). Advanced Glycation End Products Affect Growth and Function of Osteoblasts. Clin. Exp. Rheumatol..

[72] Santana R.B., Xu L., Chase H.B., Amar S., Graves D.T., Trackman P.C. (2003). A Role for Advanced Glycation End Products in Diminished Bone Healing in Type 1 Diabetes. Diabetes.

[73] Schmieder R.E. (2006). Endothelial Dysfunction: How Can One Intervene at the Beginning of the Cardiovascular Continuum?. J. Hypertens. Suppl..

[74] Libby P., Ridker P.M., Maseri A. (2002). Inflammation and Atherosclerosis. Circulation.

[75] Griendling K.K., Camargo L.L., Rios F.J., Alves-Lopes R., Montezano A.C., Touyz R.M. (2021). Oxidative Stress and Hypertension. Circ. Res..

[76] Grundy S.M., Brewer H.B., Cleeman J.I., Smith S.C., Lenfant C. (2004). Definition of Metabolic Syndrome. Circulation.

[77] Trajanoska K., Morris J.A., Oei L., Zheng H.-F., Evans D.M., Kiel D.P., Ohlsson C., Richards J.B., Rivadeneira F. (2018). Assessment of the Genetic and Clinical Determinants of Fracture Risk: Genome Wide Association and Mendelian Randomisation Study. BMJ.

